# Cost-effectiveness of 82-Rubidium PET myocardial perfusion imaging for the diagnosis of myocardial ischemia depending on the prevalence of coronary artery disease

**DOI:** 10.1186/s13550-023-00954-x

**Published:** 2023-02-08

**Authors:** Maroua Mimouni, Julie Bulsei, Meryl Darlington, Candice Estellat, François Rouzet, Fabien Hyafil, Isabelle Durand-Zaleski, the RUBIS Trial Group, Renata Chequer, Gregory Ducrocq, Toni Alfaiate, Hamza Regaieg, Jérémie Abtan, Sébastien Leygnac, Milan Milliner, Samuel Burg, Rana Ben Azzouna, Louis Potier, Cédric Laouénan, Caroline Quintin, Ronan Roussel, Gabriel Steg, Dominique Le Guludec, Emmanuel Sorbets, Laetitia Imbert, Pierre-Yves Marie, Agnès Hartemann, Gilles Montalescot

**Affiliations:** 1 DRCI-URC Eco Ile-de-France, Assistance Publique-Hôpitaux de Paris (AP-HP), Paris, France; 2 Département de Biostatistiques, Santé Publique et Informatique Médicale, Hôpital Pitié-Salpêtrière, AP-HP, 75013 Paris, France; 3 CIC-EC 1425, ECEVE UMR 1123, INSERM, 75018 Paris, France; 4 Department of Nuclear Medicine, Bichat Hospital, AP-HP, 75018 Paris, France; 5 U-1148, INSERM, Paris, France; 6 Université de Paris, 75018 Paris, France; 7 Department of Nuclear Medicine, Georges-Pompidou European Hospital, DMU IMAGINA, AP-HP, University of Paris, 75015 Paris, France; 8 U-970, INSERM, University of Paris, 75015 Paris, France; 9 Service de Santé Publique, Henri Mondor-Albert-Chenevier, Assistance Publique-Hôpitaux de Paris, Créteil, France; 10 UMR 1153 CRESS, INSERM, Paris, France; 11 UPEC, Creteil, France

**Keywords:** Cost-effectiveness, 82-Rubidium PET, SPECT, Myocardial perfusion imaging, Obstructive coronary artery disease

## Abstract

**Background:**

82-Rubidium-Positron emission tomography myocardial perfusion imaging (Rb-PET-MPI) offers higher diagnostic performance for the detection of myocardial ischemia compared to Tc-SPECT-MPI. The aim of this economic evaluation was to perform a cost-effectiveness analysis of Rb-PET-MPI versus Tc-SPECT-MPI in patients with suspected myocardial ischemia according to pretest probabilities (PTP) of obstructive coronary artery disease based on the results of the RUBIS Trial.

**Methods:**

Costs and effectiveness were calculated for all patients over 1 year and an incremental analysis of differences in costs and effectiveness in terms of diagnostic accuracy was performed. The uncertainty of the results was estimated using bootstrap. The analysis was conducted from the perspective of the French health care system with a time horizon of 12 months.

**Results:**

The average cost of a Rb-PET-MPI-based strategy for the detection of myocardial ischemia was €219 lower than a SPECT-MPI-based strategy (€1192 (± 1834) vs €973 (± 1939), *p* < 0.01). The one-year incremental cost-effectiveness ratio was negative: − €2730 (money saved per additional accurate diagnosis) in patients presenting PTP > 15% for the Rb-PET-MPI vs. Tc-SPECT-MPI strategy. Analysis of the joint distribution of costs and outcomes found that the Rb-PET-MPI strategy had a 92% probability to be dominant (cost-saving and outcome-improving).

**Conclusions:**

Rb-PET-MPI is cost-effective compared to Tc-SPECT-MPI for the detection of myocardial ischemia in patients with PTP > 15% of obstructive coronary artery disease.

***Trial registration*:**

RUBIS Trial registration: NCT01679886, Registered 03 September 2012, https://clinicaltrials.gov/ct2/show/NCT01679886.

**Supplementary Information:**

The online version contains supplementary material available at 10.1186/s13550-023-00954-x.

## Introduction

Single-photon emission computed tomography myocardial perfusion imaging (SPECT-MPI) with 99m-Technetium-radiolabeled perfusion tracers (^99m^Tc) has demonstrated its high diagnostic accuracy, prognostic value and its cost-effectiveness for the detection of myocardial ischemia [1]. Tc-SPECT-MPI has shown to be more expensive but more effective than stress echocardiography for the detection of myocardial ischemia with incremental cost-effectiveness ratios ranging from €71,930 to €168,585 per QALY gained [2]. In recent years, the diagnostic performances of Tc-SPECT-MPI benefited from technological advances but remain limited by attenuation artifacts. The diagnostic accuracy and prognostic value of CZT-SPECT derived MBF quantification is still under investigation [3, 4].

MPI with positron emission tomography with ^82^Rubidium (Rb-PET-MPI) provides several advantages over Tc-SPECT-MPI for the detection of myocardial ischemia. First, Rb-PET-MPI offers higher signal and a more accurate attenuation correction than Tc-SPECT-MPI. Second, the methods, accuracy and reproducibility for MBF quantification have been well validated for PET. Third, rest and stress Rb-PET-MPI can be acquired sequentially in 30 min with Rubidium-82 thanks to the short physical half-life of Rb reducing dramatically the time spent by patients in Nuclear Medicine department for MPI [5, 6]. Fourth, Rb-PET-MPI is associated with lower radiation exposure of patients than Tc-SPECT-MPI [7, 8]. Lastly, Rb-PET-MPI has consistently demonstrated its higher diagnostic performances than Tc-SPECT-MPI in different population of patients [6, 9]. In the RUBIS prospective clinical trial, our group has recently confirmed in 308 patients the higher diagnostic performance of Rb-PET-MPI vs. Tc-SPECT-MPI in a population of overweight patients and women for the detection of myocardial ischemia [8]. Rb-PET-MPI is clinically available since more than 20 years in North America and reimbursed, but clinical access was until now limited in Europe owing to the absence of clinically approved 82-Sr / 82-Rb generators and the lack of reimbursement for PET-MPI in most European countries. The aim of this study was to estimate the cost-effectiveness of Rb-PET-MPI compared to SPECT-MPI with 99mTc-Sestamibi in patients with different risks of coronary artery disease (CAD).

## Material and methods

### Study design

The RUBIS trial compared the diagnostic performances between Rb-PET-MPI and Tc-SPECT-MPI using CZT gamma cameras for the detection of myocardial ischemia in a population of women and overweight patients (body mass index (BMI) ≥ 25). This study was approved by the Ethics Committee of Ile-de-France VI and by the French National Agency for Medicines and Health Products (ANSM). Trial registration number NCT01679886. The method and results of RUBIS have been already published [8]. The economic analysis was carried out on three subgroups defined post hoc from the clinical analysis: low, intermediate and high pretest probabilities (PTP) of obstructive CAD, according to the ESC’s classification of PTP for obstructive CAD based on patient’s characteristics (age, sex, chest pain and dyspnea). The PTP cutoff values to classify patients are PTP < 5%, PTP between 5–15% and PTP > 15% [10]. Rb-PET-MPI and Tc-SPECT-MPI were performed successively for all patients and were compared (confirmed or rejected) to the invasive coronary angiography (ICA) with functional assessment of coronary stenosis using fractional flow reserve (FFR) measurements, which was regarded as the gold standard, for patients with abnormal examination or to the patient’s one-year clinical follow-up (ischemic events or death), in the absence of ICA. Both imaging modalities were evaluated independently from each another.

### Economic evaluation

Data for the economic evaluation were prospectively collected during the trial, in accordance with the Consolidated Health Economic Evaluation Reporting Standards (CHEERS) statement [11]. The efficacy endpoint used in the economic study was the diagnostic accuracy and was obtained from data collected in the case report form (CRF). Costs and effectiveness were assessed in all patients over 1 year and an incremental cost-effectiveness ratio of cost per additional accurate diagnostic was estimated. Bootstrapping was used to quantify uncertainty on the joint distribution of costs and outcomes, and the 1000 paired estimates of mean differential costs and diagnostic accuracy in each subgroup were reported on a cost-effectiveness plane. The study perspective was the French healthcare system and the time horizon was 12 months.

### Estimating resources and costs

Only direct costs were assessed in this economic study as recommended by the French National Authority for Health (HAS) [12]. Both hospital and non-hospital resources were considered.

Data for the Rb-PET-MPI procedure were collected by a bottom-up micro-costing analysis completed with data from the CRF and the local hospital claims database. Calculation methods and data sources used to estimate the cost of Rb-PET-MPI are detailed in Additional file 1: Tables S1, S2 and S3 online.

At the time of collecting data on resource utilization for the economic analysis, the radio-physicians carried out regular quality control routines for the Rubidium injectors. Since then, Rubidium injectors have been improved and these routines have automated, liberating human resource time for other activity. In the centers where the observations and resource data collection for the micro-costing were carried out, only ten patients were examined every week. For this analysis, taking into consideration the automation of the quality control and with the aim of maximizing efficiency, the base case taken in our analysis is 30 patients per week per injector which corresponds to the material being used for 50% of the working week.

A deterministic sensitivity analysis on the Rb-PET-MPI procedure cost was performed, varying firstly the lifetime of the CardioGen-82 generator and secondly the number of patients per generator. The SPECT-MPI procedure included a stress acquisition with injection of the Tc-sestamibi at stress (exercise, pharmacological stress with dipyridamole or combined stress) and a rest acquisition if necessary. The cost of SPECT-MPI has been evaluated in the past and the tariff was used as a proxy for production cost (see Additional file 1: Table S4 online). Data for the SPECT-MPI procedure were collected from the CRF. The total cost of each strategy (Rb-PET-MPI or Tc-SPECT-MPI) included the procedural cost, the additional test cost (ICA) if the procedural result was positive and follow-up admissions costs produced by CAD.

### Statistical analysis

The statistical analyses were performed on all patients for each of the pre-specified subgroups (low, intermediate and high PTP of myocardial ischemia). Data are described by statistical analyses (mean, standard deviation (SD) or percentage depending on the type of variable). A comparison of baseline characteristics of participants between the three subgroups was performed using the Chi-square test or ANOVA test depending on the type of variable. Cost data of Rb-PET-MPI and SPECT-MPI were compared using Student's *t*-test. Mann–Whitney's nonparametric test was performed in the case of non-normal distribution. Effectiveness was compared using Fisher’s exact test. A multiple imputation of the missing data was carried out. A *p* value less than 0.05 was considered significant. Statistical analyses were carried out using R Software (version 3.6.4).

## Results

**Fig. 1:**
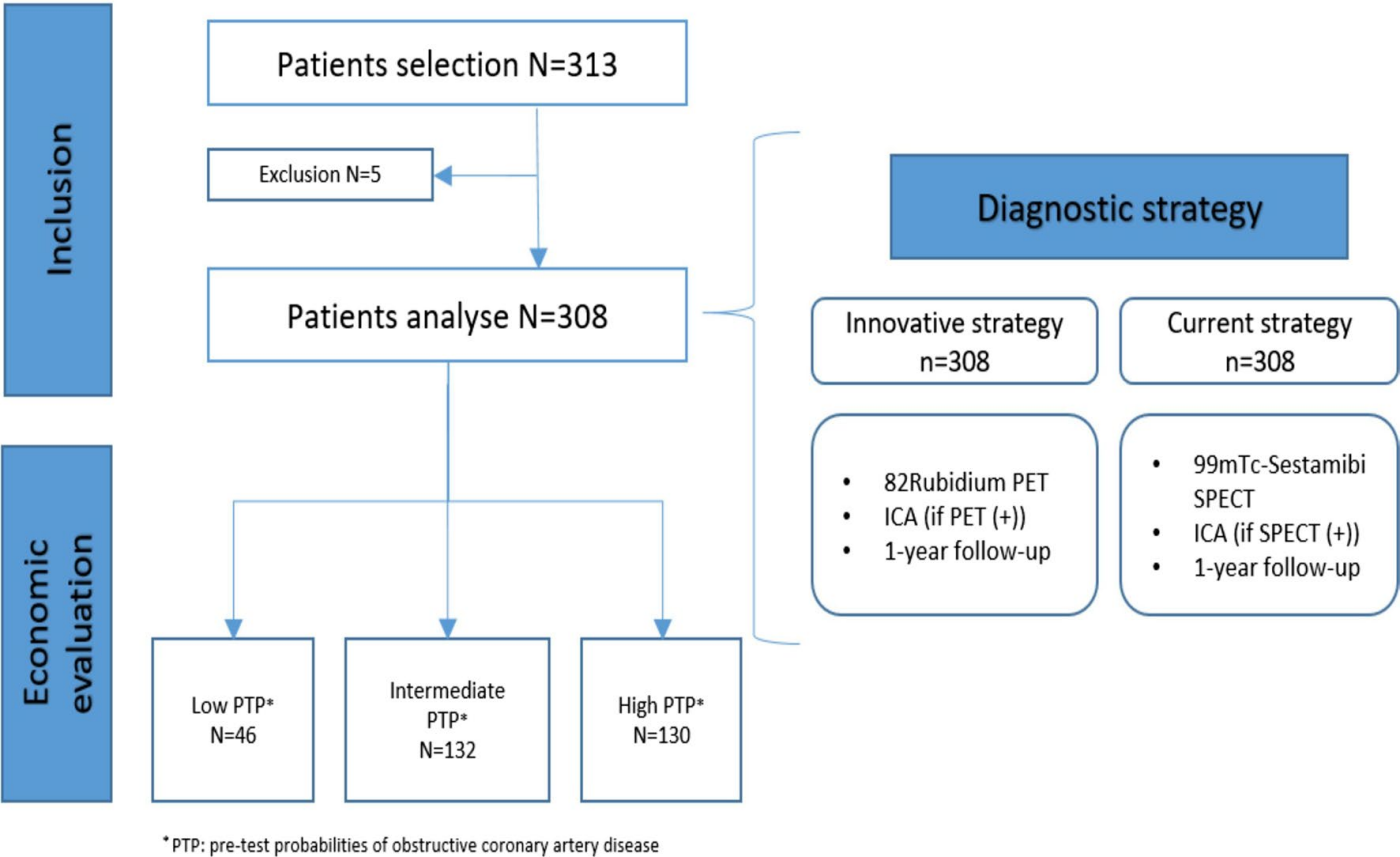
Flowchart of the economic evaluation

A total of 308 patients were included in the RUBIS trial, 46 in the low PTP subgroup, 132 in the intermediate PTP subgroup and 130 in the high PTP subgroup (Fig. 1). Baseline characteristics of RUBIS patients are presented in Table 1.

**Table 1:** Baseline characteristics of RUBIS patients

	Total patients (*N* = 308)	Patients with low PTP (*N* = 46)	Patients with intermediate PTP (*N* = 132)	Patients with high PTP (*N* = 130)	*p* value
Women, No. (%)	142 (46.1)	33 (71.7)	92 (69.7)	17 (13.1)	< 0.001
Age, mean ± SD	61 ± 9.6	49.8 ± 7.2	59.2 ± 7.8	66.2 ± 8.1	< 0.001
BMI, mean ± SD, kg/m^2^	31.8 ± 6.5	34.1 ± 7.5	32.1 ± 7.1	30.6 ± 5.1	< 0.01
Smoker (active or former), No. (%)	126 (40.9)	20 (43.5)	49 (37.1)	57 (43.8)	0.047
Active smoker, No. (%)	67 (21.8)	16 (34.8)	26 (19.7)	25 (19.2)	–
Former smoker, No. (%)	59 (19.2)	4 (8.7)	23 (17)	32 (25)	
Dyslipidemia, No. (%)	260 (84.4)	40 (86.9)	115 (87.1)	105 (80.8)	0.32
Coronary heredity, No. (%)	66 (21.4)	11 (23.9)	32 (24.3)	23 (17.7)	0.39
Hypertension, No. (%)	265 (86)	37 (80.4)	110 (83.3)	118 (90.8)	0.11
Diabetes, No. (%)	237 (76.9)	37 (80.4)	103 (78)	97 (74.6)	0.97
Type I, No. (%)	2 (0.6)	0 (0)	1 (0.8)	1 (0.8)	
Non-insulin-dependent, No. (%)	166 (53.9)	26 (56.5)	71 (53.8)	69 (53.1)	
Insulin-requiring, No. (%)	69 (22.4)	11 (23.9)	31 (23.5)	27 (20.8)	
Cardiac risk factors, mean ± SD	3 ± 0.7	3 ± 0.4	3.1 ± 0.7	3.1 ± 0.9	0.97
Chest pain, No. (%)					< 0.001
Atypical	36 (11.7)	1 (2.2)	18 (13.6)	17 (13.1)	–
Typical	46 (14.9)	1 (2.2)	18 (13.6)	27 (20.8)	–
Non-anginal	226 (73.4)	44 (95.6)	96 (72.7)	86 (66.1)	–
DF score, mean ± SD					< 0.001
Atypical chest pain	16 ± 8.1	5 ± 0	10.3 ± 6.1	22.8 ± 3.3	
Typical chest pain	56.3 ± 18.7	22 ± 0	47.5 ± 17.9	63.4 ± 15.6	
No specific chest pain	–	–	–	–	
Exertional dyspnea, No. (%)	19 (6.1)	0 (0)	8 (6.1)	11 (8.5)	0.11

### Costs

The base case is estimated from the assumption of five Rb-PET-PMI sessions scheduled per week, with an average of 6 patients per session, equivalent to 30 patients imaged per week, or to 210 patients imaged during the seven-week lifetime of the generator.

An automatic quality control (QC) of the generator was carried out before any session, eliminating time and cost of a radio-physician required for non-automatic QC. The procedural per patient cost of the Rb-PET-MPI (*n* = 308) was estimated to be €471 (± 0) for a volume of 30 patients per week. This cost included the costs of staff, supplies and imaging room which were, respectively, €65, €195 and €211. The average procedural cost of the SPECT-MPI was €757 (± 116).

In order to validate the positive test results, 47 (15%) ICA for Rb-PET-MPI and 31 (10%) ICA for Tc-SPECT-MPI were performed. The respective average costs per patient of ICA were, respectively, €191 (± 469) and €124 (± 381).

During the one-year follow-up, 16 patients (5.2%) were hospitalized at least once for a cardio-vascular event. The average cost of follow-up admissions was €311 (± 1718). The total one-year costs of Rb-PET-MPI and Tc-SPECT-MPI strategy were €973 (± 1939) and €1192 (± 1834), respectively *p* < 0.01). The one-year costs in each subgroup are presented in Table 2.

**Table 2:** One-year hospital costs of Rb-PET-MPI and Tc-SPECT-MPI diagnostic strategies in each subgroup.

	Rb-PET-MPI	Tc-SPECT-MPI	R-L [95%CI]	*p* value
	Mean (± SD)	Mean (± SD)		
Total patients	*N* = 308	*N* = 308		
Procedure	471 (± 0)	757 (± 116)	− 286 [− 299; − 273]	< 0.01
ICA admission	191 (± 469)	124 (± 381)	67 [17; 117]	0.09
Follow-up admissions	311 (± 1718)	311 (± 1718)	–	–
Total one-year cost	973 (± 1939)	1192 (± 1834)	− 219 [− 270; − 168]	< 0.01

Low PTP (< 5%)	***N* = 46**	***N* = 46**		
Procedure	471 (± 0)	762 (± 114)	− 291 [− 324; − 257]	< 0.01
ICA admission	35 (± 173)	0 (± 0)	35 [− 17; 85]	0.18
Follow-up admissions	0 (± 0)	0 (± 0)	–	–
Total one-year cost	506 (± 173)	762 (± 114)	− 256 [− 315; − 197]	< 0.01

Intermediate PTP (5–15%)	***N* = 132**	***N* = 132**		
Procedure	471 (± 0)	747 (± 124)	− 276 [− 298; − 255]	< 0.01
ICA admission	188 (± 474)	106 (± 364)	82 [7; 157]	0.033
Follow-up admissions	540 (± 2434)	540 (± 2434)	–	–
Total one-year cost	1199 (± 2715)	1393 (± 2589)	− 194 [− 271; − 118]	< 0.01

High PTP (> 15%)	***N* = 130**	***N* = 130**		
Procedure	471 (± 0)	766 (± 366)	− 294 [− 313; − 276]	< 0.01
ICA admission	249 (± 523)	185 (± 449)	64 [− 26; 155]	0.16
Follow-up admissions	189 (± 951)	189 (± 951)	–	–
Total one-year cost	909 (± 1147)	1140 (± 1042)	− 231 [− 322; − 139]	< 0.01

All costs are expressed in euro (€)

### Effectiveness

Diagnostic accuracy for the two strategies in each subgroup is presented in Table 3. In patients with low PTP and intermediate PTP, the differences in diagnostic accuracy between Rb-PET-MPI and Tc-SPECT-MPI were, respectively, − 0.04 and − 0.02 (*p* = 0.16, *p* = 0.64). In patients with high PTP, the difference was 0.08 (*p* = 0.048).

**Table 3:** Diagnostic accuracy of Rb-PET-MPI and Tc-SPECT-MPI diagnostic strategies in each subgroup (results in comparison with the reference examination)

Strategy	Number of test results				DA
	TP	FN	TN	FP	DA [95% CI]
Low PTP (< 5%) *N* = 46					
Rb-PET-MPI	0	0	44	2	0.96 [0.90–0.99]
Tc-SPECT-MPI	0	0	46	0	1
*p* value					0.16
Intermediate PTP (5–15%) *N* = 132					
Rb-PET-MPI	12	3	108	9	0.91 [0.86–0.96]
Tc-SPECT-MPI	9	6	113	4	0.92 [0.87–0.97]
*p* value					0.64
High PTP (> 15%) *N* = 130					
Rb-PET-MPI	19	6	94	11	0.87 [0.81–0.93]
Tc-SPECT-MPI	12	13	90	15	0.78 [0.71–0.85]
*p* value					0.048

*DA* diagnostic accuracy, *TP* true positive, *FN* false negative, *TN* true negative, *FP* false positive

### Incremental/decremental cost-effectiveness ratio

In the low PTP and intermediate PTP groups, Rb-PET-MPI had a lower cost and a lower effectiveness than Tc-SPECT-MPI with decremental cost-effectiveness ratios of €5888 and €12,804, respectively, indicating the additional cost of Tc-SPECT-MPI over Rb-PET-MPI for each additional accurate diagnosis at a one-year horizon. In the high PTP group, the incremental cost ratio was estimated to be − €2730 per additional accurate diagnosis, that is, the use of Rb-PET-MPI rather than Tc-SPECT-MPI would lead to an average cost-saving of €2730 for each accurate diagnosis.

### Sensitivity analysis

#### Deterministic sensitivity analysis

For six patients imaged per session and per week, with a generator lifetime of six to eight weeks, the cost of the Rb-PET-MPI procedure ranges from €449 to €498.

**Fig. 2:**
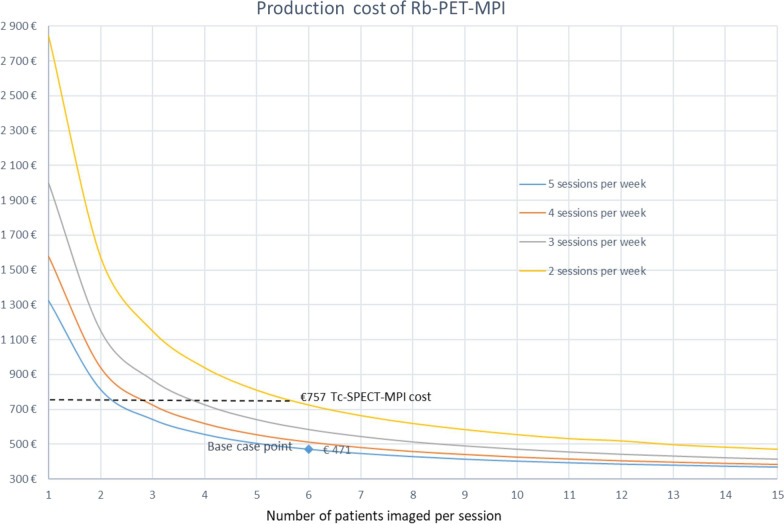
Cost of Rb-PET-MPI procedure based on the number of patients imaged per session

We evaluated the impact of the number of patients examined per session on the cost of each Rb-PET-MPI procedure (Fig. 2). The minimum number of examinations to get the Rb-PET-MPI procedure to be cost-effective was 12 per week or 105 examinations per generator over a seven-week period.

#### Probabilistic sensitivity analysis

**Fig. 3:**
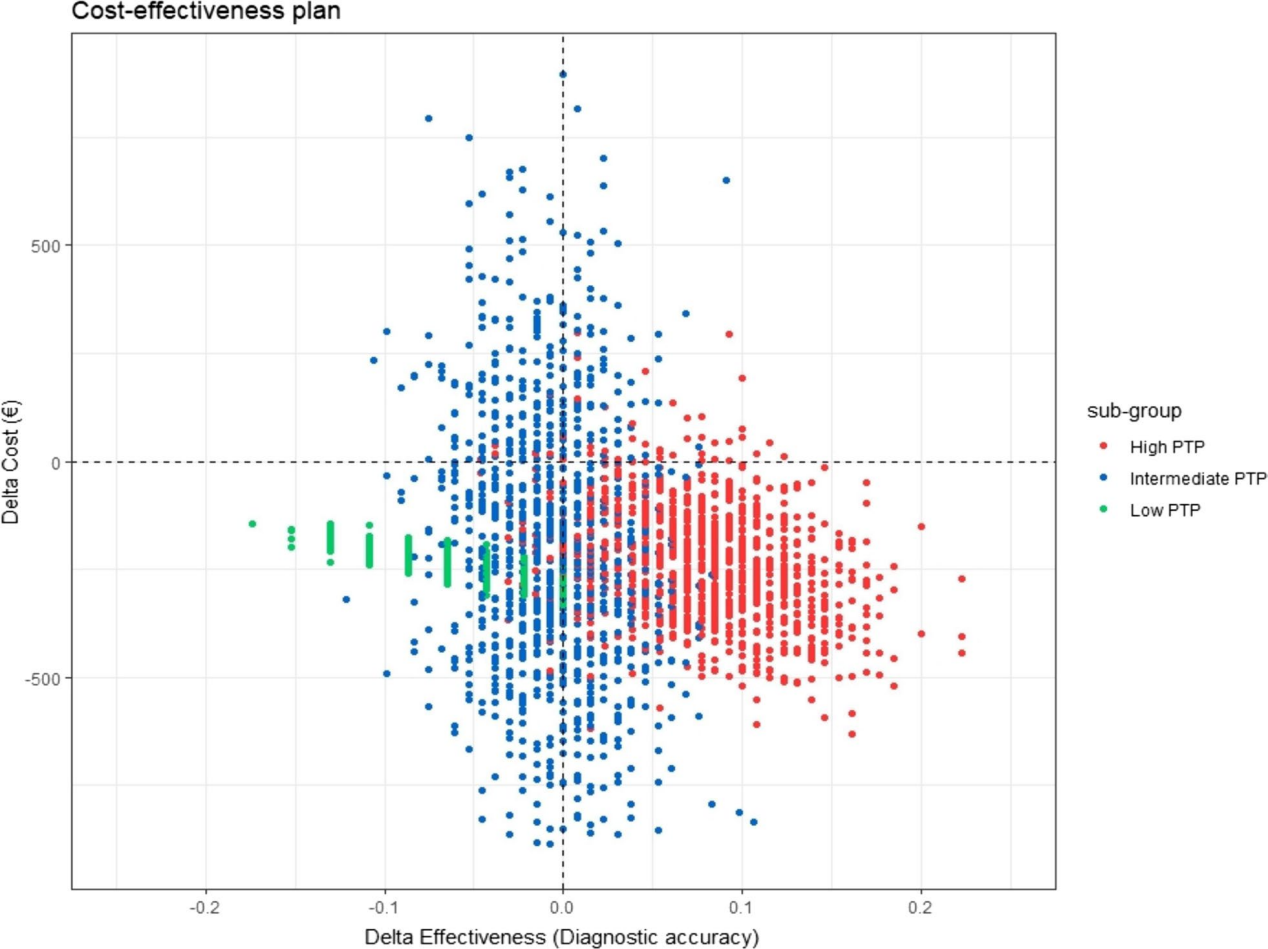
Cost-effectiveness plane: Incremental Cost-Effectiveness Ratio of Costs per additional accurate diagnosis at 12 months in each subgroup

The uncertainty on the joint distribution of costs and outcomes is presented on the cost-effectiveness plane for the three subgroups (Fig. 3).

We observed a shift of replications from the left to the right by increasing the PTP of obstructive CAD. In the low PTP subgroup, the scatter plot of the bootstrapped ICERs showed that all the replications were located in the lower left-hand quadrant indicating that the Rb-PET-MPI strategy was unlikely to be cost-effective compared to the Tc-SPECT-MPI strategy since it is cheaper but less effective.

In the intermediate PTP subgroup, the replications were scattered on the four quadrants of the plan. So, because of this uncertainty, we cannot conclude on the cost-effectiveness of the Rb-PET-MPI.

However in the high PTP subgroup, 92% of replications were located in the lower right-hand quadrant (incrementally cost-effective), indicating that the Rb-PET-MPI strategy was both cost-saving and outcome-improving (i.e., dominant) and this strategy had a high probability of being cost-effective across the entire range of willingness-to-pay values for an additional accurate diagnosis.

## Discussion

The economic evaluation of RUBIS trial estimated an average cost of €471 for the Rb-PET-MPI procedure including the costs of staff (€65), supplies (€195) and imaging room (€211) by scanning 30 patients per week or 210 patients per generator over a seven-week period. This cost was lower by €286 than the cost of SPECT-MPI with 99mTc-Sestamibi. The cost of equipment and supplies of the Rb-PET-MPI in our study was comparable to the results of a Danish costing at the Copenhagen University Hospital that estimated at €400 the cost of supplies (generator + infusion system + disposables) for 15 patients imaged per week with 82-Rubidium [5]. That is, given the fixed cost of the generator, the average cost per patient should decrease as the number of patients imaged per week increases [13]. In our study, the 82-Sr/82-Rb generator was the main cost driver representing 80% of cost of material and supplies. For example, if the number of patients scanned per week is increased to 75 (= 15 patients per day), the average cost of supplies (generator + infusion system + disposables) per patient is reduced by 48%. In addition, this new technique also saves considerable time [14]. The Tc-SPECT took typically four hours for a one-day stress/rest protocol or two separate visits of ∼ 90 min for a two-day protocol, whereas with Rb-PET-MPI 30 to 45 min for only one-day protocol were sufficient and when generator QC was automatically performed for each session.

The total costs of Rb-PET-MPI and SPECT-MPI strategies were therefore €973 (± 1939) and €1192 (± 1834), respectively (*p* < 0.01). With the volume of 30 patients imaged per week, Rb-PET-MPI was then 20% cheaper than the SPECT-MPI. These results are roughly similar to those found in an US paper of Merhige et al. evaluating the impact of Rb-PET versus SPECT on subsequent invasive procedures and outcomes, which demonstrated that Rb-PET had 30%, cost savings at 1 year compared with SPECT [15].

In the low PTP and intermediate PTP groups, Rb-PET-MPI had a lower cost and a lower effectiveness than Tc-SPECT-MPI with decremental cost-effectiveness ratios of €5888 and €12,804, respectively.

The one-year incremental cost-effectiveness ratio was negative: − €2730 (€2730 money saved per additional accurate diagnosis) in patients presenting a high PTP with the Rb-PET-MPI versus SPECT strategy indicating dominance (cheaper and more effective) of Rb-PET-MPI.

Analysis of the joint distribution of costs and outcomes showed a shift of replications from the left to the right by increasing the PTP of obstructive CAD (from less effective to more effective). The replications were evenly scattered on the four quadrants of the plan in the intermediate-risk group. This indicated that the two strategies tend to be similar. The probabilistic sensitivity analysis confirmed that the Rb-PET-MPI strategy had a 92% probability of being dominant (cost-saving and outcome-improving) in the high-risk group.

A study comparing the cost-effectiveness of exercise ECG, SPECT, PET and ICA of diagnosis of CAD showed that in a population with a high prevalence of CAD, PET had a lower cost per effect than other noninvasive tests because of its higher accuracy [16].

In a systematic review and meta-analysis, Rb-PET was demonstrated to have superior accuracy in comparison with Tc-SPECT when low likelihood risk patients were excluded and the difference in accuracy was more pronounced in favor of Rb-PET [6].

David et al. in a recent study found that the Rb-PET provided greater diagnostic accuracy in the detection of obstructive CAD relative to Tc-SPECT in extremely obese patients (diagnostic accuracy: Rb-PET 86.3% versus Tc-SPECT 64.9%, *p* = 0.02) [17].

For patients with high PTP of obstructive CAD our results support the greater accuracy of Rb-PET-MPI versus Tc-SPECT-MPI (diagnostic accuracy: Rb-PET 87% vs. Tc-SPECT 78%, *p* = 0.05) and confirm that Rb-PET-MPI was potentially cost-effective in detecting myocardial ischemia in patients with high PTP of obstructive CAD.

The costs of the procedures were lower in our study than in US studies previously published, with cost per scan ranging from €1000 to 2000 [15, 16]. Costs in Europe tend to be lower ranging from €260–450 for SPECT to €1000 for PET [18].

### Limitations of the study

First, in the low risk subgroup, we cannot conclude on the effectiveness of Rb-PET-MPI compared to Tc-SPECT-MPI because of the small number of patients included and the absence of positive cases. Two patients were falsely classified as positive with Rb-PET which could lead to overtreatment.

Second, the cost results of this economic study only concern the French environment and are not directly applicable in other countries. Third, invasive coronary angiography and FFR were used as gold standards for the evaluation of the diagnostic performance of MPI with SPECT and PET, but offer only to assess the hemodynamic impact of epicardial coronary stenosis on hyperemic blood flow and do not provide accurate measurements of regional myocardial perfusion.

## Conclusions

Rb-PET-MPI estimated cost was lower than the cost of SPECT-MPI with 99mTc-Sestamibi. The cost of Rb-PET is highly dependent on an efficient use of the materiel in terms of the number of weekly sessions and the number of patients per session.

In this study, we demonstrated that Rb-PET was cost-effective compared to Tc-SPECT for the detection of myocardial ischemia in patients with PTP > 15% of obstructive CAD.

## Supplementary Information

**Additional file 1. Table S1.** Calculation method, data sources and valuation used to calculate the cost of Rb-PET-MPI (procedural and follow-up costs). **Table S2.** Hourly cost of human resources of Rb-PET-MPI intervention. **Table S3.** Unit cost of supplies and drugs used on Rb-PET-MPI procedure. **Table S4.** Resources used in Tc-SPECT-MPI procedure

## Data Availability

The datasets generated during and/or analyzed during the current study are available from the corresponding author on reasonable request.

## Abbreviations

BMI: Body mass index
CHEERS: Consolidated Health Economic Evaluation Reporting Standards
CAD: Coronary artery disease
DF: Diamond-Forrester
FFR: Fractional flow reserve
HAS: French National Authority for Health
ICA: Invasive coronary angiography
ANSM: National Agency for Medicines and Health Products
PET-MPI: Positron emission tomography myocardial perfusion imaging
PTP: Pretest probabilities
QC: Quality control
SPECT-MPI: Single-photon emission computed tomography myocardial perfusion imaging
^99m^Tc: 99M-Technetium
Rb: 82-Rubidium
