# Supplementary material for: Cost-effectiveness of 82-Rubidium PET myocardial perfusion imaging for the diagnosis of myocardial ischemia depending on the prevalence of coronary artery disease
Source: EJNMMI Res. 2023 Feb 8;13:9. doi: 10.1186/s13550-023-00954-x (PMC9908793; doi:10.1186/s13550-023-00954-x)
Supplement: Supplementary file 1 — Additional file 1. Table S1. Calculation method, data sources and valuation used to calculate the cost of Rb-PET-MPI (procedural and follow-up costs). Table S2. Hourly cost of human resources of Rb-PET-MPI intervention. Table S3. Unit cost of supplies and drugs used on Rb-PET-MPI procedure. Table S4. Resources used in Tc-SPECT-MPI procedure [file 13550_2023_954_MOESM1_ESM.pdf]

# Cost-effectiveness of 82-Rubidium PET myocardial perfusion imaging for the diagnosis of myocardial ischemia depending on the prevalence of coronary artery disease

Maroua Mimouni, Julie Bulsei, Meryl Darlington, Candice Estellat, François Rouzet, Fabien Hyafil, Isabelle Durand-Zaleski, on behalf of the RUBIS Trial Group

## SUPPLEMENTARY MATERIALS

**Table S1.** Calculation method, data sources and valuation used to calculate the cost of Rb-PET-MPI (procedural and follow-up costs)

| | Calculation method | Sources of data | Valuation |
|---|---|---|---|
| **Human resources** | Cost of staff = Σ (hourly personnel cost x time of attendance x number of people) | The number and type of staff, the duration of the interventions and the time spent in the radiology room are collected during microcosting. | The hourly personnel cost is calculated on the basis of the salary and charges for the average grade of each type of staff [1] (Table S2).<br>We consider that a person worked 1,607 hours during the year.[2] |
| **Material, supplies and drugs** | Cost of consumables = Σ (purchase price of a consumable x number of consumables) | The different consumables are collected during microcosting | The unit prices of generator, infusion system and other supplies are provided by the company A.A.A (Advanced Accelerator Applications) have been valued at their purchase price (Table S3).<br>The unit prices of drugs have been valued at their purchase price. (Table S3). |
| **Intervention room** | Cost of the intervention room = room cost per hour x room occupancy | The duration of the procedure is collected during microcosting. | The cost of the radiology room is extracted from the 2012 cost accounting of Beaujon hospital.<br>We consider that during this year, the intervention room operated 8 hours a day for 250 working days. |

| | | | |
|---|---|---|---|
| **ICA admission** | Cost of hospital stay = (cost of the stay for the DRG / average length of stay observed in DRG) x Length of Stay | The length of stay of each patient and the Diagnosis-Related Group (DRG) are collected from the hospital billing database | The cost of the hospitalizations is valued using GHS cost (ATIH 2013).[3] |
| **Follow-up admissions** | hospitalizations cost = ∑ (GHS tariff) | The length of stay of each patient and the DRG are collected from the hospital billing database. | The cost of the hospitalizations is valued using GHS cost (ATIH 2013).[3] |

[1] B. Hagenmuller, L. Geoffroy, E. Guyader, J. Lignon, D. Rouquette, M. Beudaert, B. de la Chapelle, B. Chenais, A. Desmoulières, and P. Thurat, Guide pour le suivi de la masse salariale dans les établissements publics de santé. Ministère de l’emploi et de la solidarité, 06-Jul-2001.
[2] Code du travail - Article L3123-1. Available online:https://www.legifrance.gouv.fr/codes/article_lc/LEGIARTI000033020098. (Accessed on 12-Apr-2020).
[3] Financement et données financières, ATIH. Available online: http://www.atih.sante.fr/tarifs-mco-et-had. (Accessed on 12-Apr-2020).

**Table S2.** Hourly cost of human resources of Rb-PET-MPI intervention

| Staff | Total employer cost inclusive of taxes (€) | Cost per hour (€) |
|---|---|---|
| Radiology technician | 51,468 | 32.0 |
| Senior physician | 137,494 | 85.6 |

**Table S3.** Unit cost of supplies and drugs used on Rb-PET-MPI procedure

| Material, supplies and drugs | | Unit price (€) | Price (€)/patient |
|---|---|---|---|
| One use only | | | |
| CardioGen-82 accessories | Patient Administration Sets (25sets- 1per patient) | 480 | 19.20 |
| | Assembly Extension Tubing (15 per box- 1per patient) | 72 | 4.80 |
| DIPYRIDAMOLE 10 MG/2 ML | | 0.58 | 0.58 |
| AMINOPHYLLINE 250 MG/10 ML | | 1.63 | 1.63 |
| Seven-week use (lifetime generator) : Base case | | | |
| CardioGen-82 Generator | | 32,670 | 155.57 |
| Infusion System | | 1,800 | 8.57 |

| | | | |
|---|---|---|---|
| CardioGen-82 accessories | Accessory Pack | 120 | 0.57 |
| | Waste Bottle | 36 | 0.17 |
| | Elution Test Vials | 84 | 0.40 |
| | Elution Test System | 720 | 3.43 |
| | Elution Vial Shield | 42 | 0.20 |
| | Thermal Printer paper | 30 | 0.14 |

**Table S4.** Resources used in Tc-SPECT-MPI procedure

| **Tc-SPECT-MPI** | **French Heathcare reimbursement (€)** | **Unit price (€)** |
|---|---|---|
| Myocardial perfusion tomoscintigraphic acquisition | 319.4 | _ |
| Pharmacological stress (dipyridamole) | _ | 0.6 |
| Stress test on a treadmill or ergometric bicycle | 76.8 | _ |
| Resting Myocardial perfusion tomoscintigraphic acquisition | 316.8 | _ |
| Radiopharmaceutical (99mTc-Sestamibi) | _ | 45.5 |
